# Molecular underpinnings of clinical disparity patterns in African American vs. Caucasian American multiple myeloma patients

**DOI:** 10.1038/s41408-019-0177-9

**Published:** 2019-02-04

**Authors:** Dickran Kazandjian, Elizabeth Hill, Malin Hultcrantz, Evan H. Rustad, Venkata Yellapantula, Theresia Akhlaghi, Neha Korde, Sham Mailankody, Alex Dew, Elli Papaemmanuil, Irina Maric, Mary Kwok, Ola Landgren

**Affiliations:** 1 Myeloma Program, Lymphoid Malignancies Branch, Center for Cancer Research, National Cancer Institute, National Institutes of Health, Bethesda, MD USA; 2 Myeloma Service, Department of Medicine, Memorial Sloan Kettering Cancer Center, New York City, NY USA; 3 Epidemiology and Biostatistics, Memorial Sloan Kettering Cancer Center, New York City, NY USA; 4 Department of Hematology-Oncology, Walter Reed National Military Medical Center, Bethesda, MD USA; 5 Hematology Section, Department of Laboratory Medicine Clinical Center, National Institutes of Health, Bethesda, MD USA

## Abstract

Caucasian Americans (CA) compared with African Americans (AA) have a twofold increased incidence of multiple myeloma (MM) and have an earlier age of diagnosis. However, there is sparse information regarding underlying biological differences across racial/ethnic groups. We characterized genetic alterations using a targeted next-generation sequencing assay called myTYPE, developed at MSKCC, allowing capture of somatic mutations, IgH translocations, gains/losses, and hyperdiploidy. Samples were obtained from the NIH Plasma Cell Dyscrasia Racial Disparity Cohort. In total, 68 patient samples were successfully sequenced and manually curated based on well-established databases. Of the 68 patient samples (47 CA, 21 AA), 84% had at least one type of genomic alteration. Importantly, the IgH translocation, t(11;14), was observed more frequently in the AA group (0 vs. 29%, *p* = 0.001). Known oncogenic somatic non-synonymous mutations were found in 18 genes and indels in 2 genes. *KRAS* mutations were the most common mutation found in 16% of patients followed by *NRAS* and *BRAF* mutations. *TP53* somatic mutations appeared to be more common in CA but lacked significance. This proof-of-principle study indicates the presence of varying underlying tumor biology between racial groups and supports the need of future prospective trials to capture these molecular characteristics.

## Introduction

Despite advancements in the understanding and treatment of multiple myeloma (MM), a racial disparity in clinical presentation and outcomes remain. Compared with Caucasian Americans (CA), African Americans (AA) matched for socioeconomics, age, and gender have a twofold increased incidence of MM, have an earlier average age at diagnosis by 5–10 years, and have gained less benefit from the advent of novel agents in the last decade^1,2^. These differences have not been shown to be attributable to disparities in access to medical care. In addition, over the past decade, improvements in survival with the introduction of proteasome inhibitors and immunomodulatory agents is predominantly observed in CA. Costa et al.^3^ observed improvements in 10-year relative survival rates (RSRs) in all racial groups < 65 years of age and no improvements for either racial group over 75 years of age. In patients between the ages of 65 and 74 years, CA had an improvement in 10-year RSRs but AA did not. Moreover, although it has been noted that AA have an increased myeloma-related mortality rate, this is in fact a reflection of the increased incidence of MM in AA rather than worse prognosis. In a pivotal study of 30,000 patients, the authors concluded that AA appear to have a better prognosis compared with CA^4^.

The variation in clinical course suggests an underlying molecular heterogeneity between races. Despite the increased frequency of MM among AA, most of the known molecular data and association with clinical outcomes, including traditional fluorescence in situ hybridization (FISH)/cytogenetics and newer NGS methods have been derived from CA cohorts^5–8^. At this time there is no single unifying genetic or genomic alteration known to cause MM but there are multiple alterations frequently identified. Approximately half of MM genomes are hyperdiploid (gain of an additional odd numbered chromosomes)^9,10^. Most of the non-hyperdiploid MM cases harbor a translocation involving the immunoglobulin heavy-chain (IgH) gene located on chromosome 14^9,10^. These genetic lesions are thought to be primary events, as they are also found in the precursor state, monoclonal gammopathy of undetermined significance (MGUS)^11^. In ~10% of cases, both aberrations co-occur^12,13^. In general, hyperdiploid MM is associated with an improved prognosis compared with MM cases with an IgH translocation, except for the cyclin D translocations (t(6;14) and t(11;14)), which are considered neutral^14,15^. The five most frequent translocations in descending order are t(11;14), t(4;14), t(14;16), t(14;20), and t(6;14)^15,16^. Based on karyotyping and interphase FISH, t(4;14), t(14;16), and t(14;20) have been identified as high-risk primary genetic events, along with the secondary/tertiary events of deletion 17p, deletion 1p32, and 1q gains^9,14^. The genetic heterogeneity of myeloma is reflected in the variety of genetic hits including secondary translocations, copy number variants (CNVs), and somatic oncogenic mutations^17^.

To improve our understanding of the underlying biological mechanisms of the racial disparity in patients with MM, this study used a targeted NGS assay termed myTYPE developed at Memorial Sloan Kettering Cancer Center. myTYPE was specifically developed to target genomic aberrations known to occur in patients with MM^18,19^. The myTYPE assay is designed to capture known IgH translocations, hyperdiploidy, CNVs, and somatic mutations in 120 frequently mutated genes in MM. Using this specific assay we investigated the differences in somatic mutations, translocations, and chromosomal gains/losses between CA and AA MM patients.

## Methods

### Patients and techinical assays

Bone marrow clot sections were obtained from the National Institutes of Health Plasma Cell Dyscrasia Racial Disparity Cohort. A total of 91 pretreatment baseline samples from patients with newly diagnosed MM (NDMM) underwent DNA extraction, 81 samples met DNA quality control (QC) and purity criteria, and underwent NGS library preparation. Of these, 68 (47 CA, 21 AA) patient samples passed all QC measures for sequencing. In the myTYPE assay, baits were designed to capture the entire IgH locus (where the majority of the canonical chromosome 14 breakpoints occur) and the partner chromosome, genome-wide single-nucleotide polymorphisms for hyperdiploidy, and other CNVs as well as exons of 120 frequently mutated genes in MM. With this design, myTYPE detects the IGH translocations and the partner chromosome regardless of which the partner is. In addition, myTYPE detects hyperdiploidy, arm-level chromosomal gains and losses, as well as the most common and relevant somatic mutations. The target regions from bone marrow clot section samples were amplified and then sequenced using 126 base-paired end reads using Illumina HiSeq with a mean target depth of 413.5×. Data were analyzed using validated bioinformatic algorithms (Supplementary Methods). Race was determined from patient self-reporting. Fisher’s exact test was used to calculate two-tailed *p*-values for differences between CA and AA groups. The Bonferroni method was used to correct for multiple testing and control the family-wise error rate at <0.05. This resulted in a significance threshold of *p* < 0.0015 for each comparison.

## Results

**Fig. 1:**
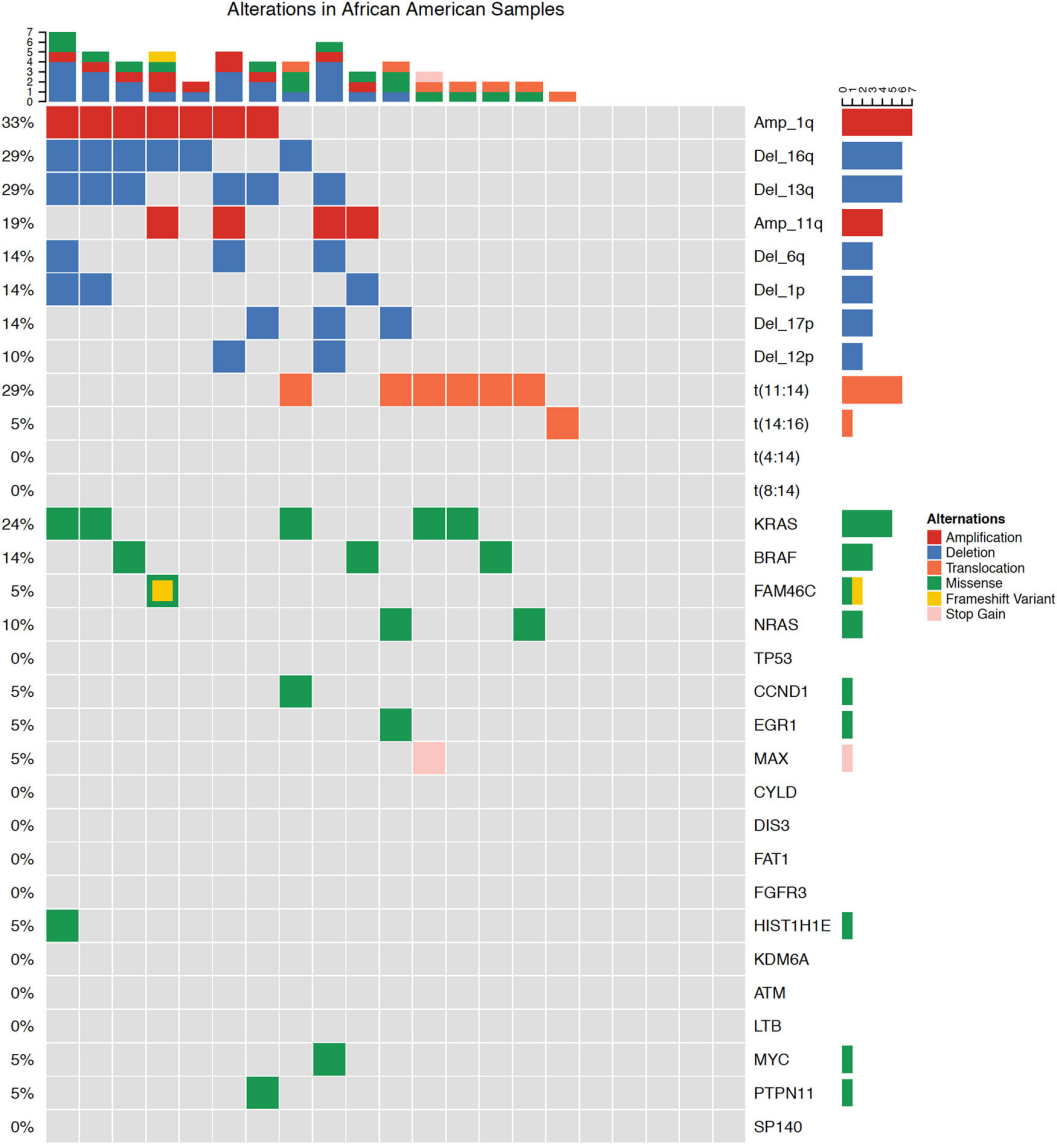
Genetic alterations in African American (AA) patient samples. Sixteen of 21 AA patients myeloma samples had either an amplificant or deletion, IgH translocation, insertion/deletion, or oncogenic missense mutation. KRAS mutations were the most frequently mutated gene. Compared with Caucasian Americans, AA were enriched for t(11;14)

**Fig. 2:**
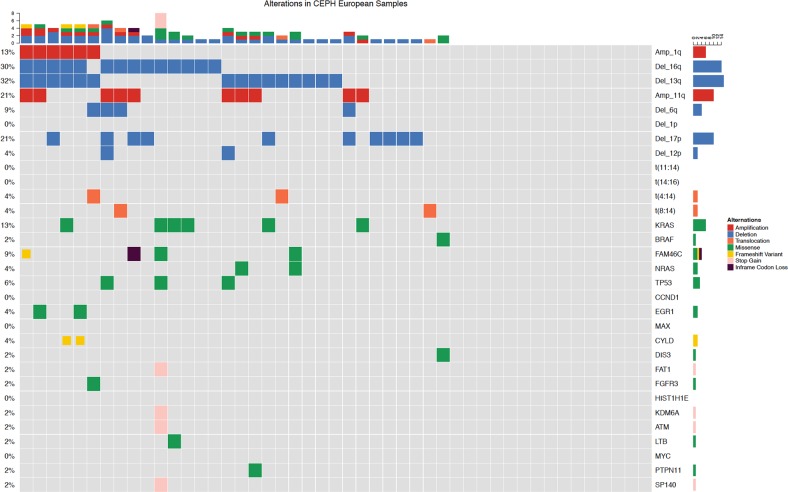
Genetic alterations in Caucasian American (CA) patient samples. Thirty-two of 47 CA patients’ myeloma samples had either an amplificant or deletion, IgH translocation, insertion/deletion, or oncogenic missense mutation. KRAS mutations were the most frequently mutated gene. Compared with African Americans, CA had an increased frequency of TP53 oncogenic mutations but did not reach statistical significance

Of the 68 patient baseline samples sequenced (47 CA, 21 AA), 57 patients (87%) had at least one genomic alteration (i.e., hyperdiploidy, translocations, chromosomal gains/losses, indels, or somatic non-synonymous mutations). Of these, 20 (95%) were from AA and 37 (79%) from CA patient samples (Figs. 1 and 2). Putative oncogenic mutations and indels (insertions and deletions) were observed in 19 oncogenic genes (Table 1). *KRAS* mutations were most common, identified in 16% of patients (13% CA, 24% AA*). NRAS* (4% CA, 10% AA) and *BRAF* (2% CA, 14% AA) mutations were the second most common. *BRAF* mutations were all V600E non-synonymous mutations and statistically trended to be more common in AA (*p* = 0.08). *TP53* somatic mutations also appeared to be more common in CA (6%) than AA (0%); however, this difference did not reach statistical significance. Oncogenic mutations were also observed in *FAM46C*, *EGR1*, *PTPN11*, *ATM*, *CCND1*, *DIS3*, *FAT1*, *FGFR3*, *HIST1H1E*, *KDM6A*, *LTB*, *MAX*, *MYC*, and *SP140*. Indels (frameshift and in-frame variants) were identified in *FAM46C* and *CYLD*. In 11 of the patient samples, no genomic alterations were observed.

**Table 1:** Frequency of oncogenic somatic mutations and indels and comparison between race

Gene	Type	ALL, *n* (*N* = 68)	CA, *n* (*N* = 47)	% (*n/N*)	AA, *n* (*N* = 21)	% (*n/N*)	*P*-value*
*KRAS*	NSM	11	6	12.8	5	23.8	0.295
*FAM46C*	NSM, IFCL, FSV	6	4	8.5	2	9.5	1
*BRAF*	NSM	4	1	2.1	3	14.3	0.084
*NRAS*	NSM	4	2	4.3	2	9.5	0.582
*EGR1*	NSM	3	2	4.3	1	4.8	1
*TP53*	NSM	3	3	6.4	0	0	0.547
*CYLD*	FSV	2	2	4.3	0	0	1
*PTPN11*	NSM	2	1	2.1	1	4.8	0.526
*ATM*	SG	1	1	2.1	0	0	1
*CCND1*	NSM	1	0	0	1	4.8	0.309
*DIS3*	NSM	1	1	2.1	0	0	1
*FAT1*	SG	1	1	2.1	0	0	1
*FGFR3*	NSM	1	1	2.1	0	0	1
*HIST1H1E*	NSM	1	0	0	1	4.8	0.309
*KDM6A*	SG	1	1	2.1	0	0	1
*LTB*	NSM	1	1	2.1	0	0	1
*MAX*	SG	1	0	0	1	4.8	0.309
*MYC*	NSM	1	0	0	1	4.8	0.309
*SP140*	SG	1	1	2.1	0	0	1

*AA* African Americans; *CA* Caucasian Americans; *FSV* frameshift variant; *IFCL* in-frame codon loss; *NSM* non-synonymous mutation; *SG* stop gain

*Two sided Fisher’s test *p* = 0.05

Four different translocations involving IgH were identified in patients (Table 2). The t(11;14) occurred significantly more frequently in the AA group (29% vs. 0, *p* = 0.0005). This difference remained significant when adjusting for multiple testing. The incidence of t(4;14), t(8;14), and t(14;16) were not significantly different between the CA and AA groups. Chromosomal gains and losses were identified in many patients and, as expected, approximately half the patients had hyperdiploid myeloma (Table 2). Moreover, del(1p) was observed more commonly in the AA population (0 vs. 14%, *p* = 0.037) as well as amp(1q) (13% vs. 33%, *p* = 0.091); however, neither remained significant when adjusting for multiple testing. There were no other significant differences between racial groups including patients with hyperdiploid genomes, del(17p), or del(13q).

**Table 2:** Frequency of IgH translocations, hyperdiploidy, and copy number variations and comparison between race

Gene	Type	ALL, *n* (*N* = 68)	CA, *n* (*N* = 47)	% (*n/N*)	AA, *n* (*N* = 21)	% (*n/N*)	*P*-value*
4;14	Translocation	2	2	4.3	0	0	1
8;14	Translocation	2	2	4.3	0	0	1
11;14	Translocation	6	0	0	6	28.6	0.0005
14;16	Translocation	1	0	0	1	4.8	0.309
Hyperdiploidy	Chromosomal gain	34	24	51.1	10	47.6	1
amp(1q)	Amplification	13	6	12.8	7	33.3	0.091
del(1p)	Amplification	3	0	0	3	14.3	0.027
del(13q)	Amplification	21	15	31.9	6	28.6	1
del(17p)	Amplification	13	10	21.3	3	14.3	0.74

*AA* African Americans; *CA* Caucasian Americans

*Two sided Fisher’s test *p* = 0.05

Given the striking observation that t(11;14) was more common in AA patient samples, IgH translocation data were downloaded from the Multiple Myeloma Research Foundation (MMRF) CoMMpass (Relating Clinical Outcomes in MM to Personal Assessment of Genetic Profile) Study (research.themmrf.org) to analyze and determine whether racial differences in t(11;14) were observed in a larger cohort of patients^20^. To the best of our knowledge, racial differences in translocations in this cohort had not been previously reported. Based on validated racial classification previously reported, 658 samples were evaluable for chromosome 14 translocations (112 AA; 546 CA). No statistically significant differences were observed between races including t(11;14) (Table 3)^8^.

**Table 3:** Frequency of IgH translocations in the CoMMpass Cohort

Translocation	ALL, *n* (*N* = 658)	CA, *n* (*N* = 546)	% (*n/N*)	AA, *n* (*N* = 112)	% (*n/N*)	*P*-value*
4;14	79	67	12.3	12	10.7	0.751
6;14	7	7	1.3	0	0.0	0.609
8;14	91	75	13.7	16	14.3	0.881
11;14	138	115	21.1	23	20.5	1
14;16	26	20	3.7	6	5.4	0.386
14;20	10	7	1.3	3	2.7	0.386

Abbreviations: *AA* African Americans; *CA* Caucasian Americans

*Two sided Fisher’s test *p* = 0.05

## Discussion

These results suggest the presence of genetic heterogeneity between MM racial groups. Our results are consistent with the existing MM molecular literature including the observation that *KRAS*, *NRAS*, *DIS3*, and *TP53* are commonly mutated genes in MM^8^. As described in many malignancies, in MM, *TP53* mutations indicate a poor prognosis and shorter survival; however, the effects of other mutations are not well characterized^21,22^. We observed *TP53* mutations more frequently in CA, which is consistent with the findings from a recent study that also observed significantly higher *TP53* mutation rates among CA MM cases^8^. Located on chromosome 17p13.1, *TP53* encodes for p53 tumor suppressor protein mediating multiple cell cycle pathways including apoptosis, cell cycle arrest, and inhibition of angiogenesis^23^. In MM, *TP53* mutations are a rare occurrence at diagnosis; however, the incidence increases as patients are treated. It is often associated with poor prognosis and accounts for a significantly lower survival rate^24^. This finding suggests potentially one etiology for the worse prognosis observed in CA^4^. Furthermore, this may have important clinical implications in terms of targeted drug development. For example, compounds in various stages of development including MDM2 inhibitors, focus on restoring wild-type p53 activity^25^. A few of these agents have proceeded to first-in-human phase 1 interventional MM clinical trials^26^.

Importantly, in our cohort, t(11;14) was found to be significantly more frequent in the AA group (*p* = 0.0005)*.* t(11;14) is a frequent translocation in MM found in about 15–20% of patients^10,27,28^. Recently, the Mayo Clinic group using traditional FISH methods observed a similar association with t(11;14) in their AA cohort^29^. They evaluated 881 patients with monoclonal gammopathies and found that the probability of having t(11;14) (or t(14;16)/t(14;20) was significantly higher in the 120 patients with highest AA ancestry (≥80%) compared with individuals with lowest levels of AA ancestry. This finding helps to confirm that our results are not random, and that NGS methods can be used to confirm traditional FISH findings. The t(11;14)(q13;q32) results in upregulation of cyclin D1, thus promoting cell cycle progression^10^. Most data support that the presence of t(11;14) is associated with neutral or standard prognostic risk, and that it may confer improved survival and response to treatment compared with the other commonly observed IgH translocations^10,28,30,31^. However, a few smaller studies suggest that patients with MM harboring t(11;14) may not have the same prognosis as patients with other standard risk features^32^. Interestingly, recent work suggests that AA patients with t(11;14) showed a trend toward shorter median progression-free survival (PFS) compared with AA without the presence of t(11;14); however, t(11;14) did not impact PFS in non-AA patients^33^. More importantly, this genetic alteration also has significant implications for drug development. More recently, it has been observed that patients with this translocation are much more likely to respond to BLC-2 inhibitors, and that this genotype is associated with increased expression of the anti-apoptotic protein BCL-2 compared with pro-apoptotic family members. For example, the BCL-2 inhibitor, venetoclax, as monotherapy is associated with a response rate of 40% in patients with t(11;14) compared with 21% in all comers^34^. In short, we were unable to confirm our finding in the CoMMpass cohort; however, the confirmatory finding in the Mayo AA cohort reinforces this finding, which has important implications in the AA population and precision drug development.

Deletion of chromosome 1p, (del(1p)), which is associated with a poor prognosis, in our cohort, appeared to also be more frequent (*p* = 0.037) along with amp(1q) in AA; however, these were not significant after adjusting for multiple testing and no differences were observed in the CoMMpass cohort^8,35^. Although t(11;14) is thought to be a primary event as it is observed in the early precursor state of MGUS, del(1p) is thought to be a secondary event further driving MM clonal evolution^36^. Therefore, this finding may be biased by the timing of when patients were diagnosed.

In addition to risk prognostication, the differences in somatic mutations among races may have significant implications in the development of targeted therapies. For example, the *BRAF*^*V600E*^ mutation is a frequent and well-described driver mutation in melanoma and hairy cell leukemia with approximate response rates of 50–60% and 96–100%, respectively, to *BRAF* inhibition with tyrosine kinase inhibitors^37–39^. Interestingly, *BRAF* mutations occur in ~5% of MM cases with tumors that respond to tyrosine kinase inhibition^40,41^. Albeit a rare driver mutation in MM, it is an important druggable target. Interestingly, we observed a higher rate of *BRAF*^*V600E*^ mutations in AA (14%) compared with CA (2%); however, this finding was not statistically significant and was not observed in the independent CoMMpass cohort.

Our findings along with other works suggest that the incidence of the various prognostic primary genetic events is not significantly different between races (e.g., t(4;14) and hyperdiploidy). Rather, the differences between races are predominantly events known to occur later in disease evolution. The molecular pathology of MM changes overtime and multiple clonal competitions occur in the cancer cell population through branching evolution^36^. Clonal and sub-clonal evolution occurs in the context of pressures present in the tumor micro-environment including treatment effect creating a branching nonlinear pathway of multiclonal MM development^5^. Based on this, one might speculate that the primary pathogenic events are similar across races, whereas the ensuing disease evolution follows slightly different trajectories, shaped by inter-racial differences in tumor–host interactions^42^.

This study is limited by the small number of patients. However, it expands upon the limited molecular data from AA with MM. We plan to further study and expand on these findings by examining the genetic alterations present and associated clinical outcome differences between races in patients with smoldering MM and comparison with NDMM. Our current actively enrolling study Carfilzomib, Lenalidomide, and Dexamethasone in High Risk Smoldering Multiple Myeloma will be the vehicle to aid in answering these important questions and thus far has shown impressive results at interim analysis (https://clinicaltrials.gov/ct2/show/NCT01572480)^43,44^. This information will add to our knowledge of the clonal evolution of MM, prognostic value of genetic data, and elucidate potential differences in smoldering myeloma compared with NDMM in terms of race.

## Conclusions

The findings of this work significantly contribute to the understanding of molecular differences between races in MM, in a relative knowledge desert. These findings argue for more enrichment of AA patients in prospective MM treatment trials and characterization of molecular profiles.

## Supplementary information

supplemental_methods-revised-clean
