# Supplementary material for: Molecular underpinnings of clinical disparity patterns in African American vs. Caucasian American multiple myeloma patients
Source: Blood Cancer J. 2019 Feb 4;9(2):15. doi: 10.1038/s41408-019-0177-9 (PMC6361959; doi:10.1038/s41408-019-0177-9)
Supplement: Supplementary file 1 — supplemental_methods-revised-clean [file 41408_2019_177_MOESM1_ESM.docx]

**Molecular Underpinnings of Clinical Disparity Patterns in African American versus Caucasian American Multiple Myeloma Patients**

## Supplemental methods

**myTYPE Data Generation**

All 68 tumor samples and 16 unmatched constitutional samples were subjected to a targeted custom capture approach using myTYPE. myTYPE is a custom capture panel designed which includes the entire IgH locus (where the majority of the canonical chromosome 14 breakpoints occur), genome wide single nucleotide polymorphisms (SNPs) for hyperdiploidy and other CNVs as well as exons of 120 recurrently mutated genes in MM. myTYPE thus detects chromosome 14 translocations and the partner chromosome, hyperdiploidy, arm level gains and losses as well as somatic mutations in 120 frequently mutated and relevant genes in multiple myeloma.

**Short Read Alignment**

Short insert paired-end reads were aligned to the GRCh37 reference human genome with 1000 genomes decoy contigs using BWA-mem^1^. After sequencing we obtained a median of 33.6 million 126 bp paired-end reads per sample. After alignment, we obtained a median mean bait coverage of 413.5X per sample (Q1:330;Q3:600.4).

**Somatic Mutation Calling**

Substitutions

Single base substitutions were called using cgp CaVEMan^2^. As described previously^3^, the algorithm compares sequence data from each tumour sample albeit with an unmatched non-cancerous sample and calculates a mutation probability at each genomic locus. To improve specificity, a number of post-processing filters were applied as follows:

1. At least a third of the alleles containing the mutant must have base quality >= 25.

2. If mutant allele coverage >= 10X, there must be a mutant allele of at least base quality 20 in the middle 3rd of a read. If mutant allele coverage is < 10X, a mutant allele of at least base quality 20 in the first 2/3 of a read is acceptable.

3. The mutation position is marked by <3 reads in any sample in the unmatched normal panel.

4. The mutant allele proportion must be >5 times than that in the unmatched normal sample (or it is zero in the unmatched normal).

5. If the mean base quality is <20 then less than 96% of mutations carrying reads are in one direction.

6. Mutations within simple repeats, centromeric repeats, regions of excessive depth (<https://genome.ucsc.edu/>) and low mapping quality were excluded.

Additional unmatched normal flagging of variants was performed using a set of unmatched normal samples. Mutations that were detected in >5% of the unmatched normal normal panel at >=5% mutant allele burden were excluded.

Variant annotation was done in Ensembl v74 using VAGrENT^4^.

Small insertions and deletions

Small somatic insertions and deletions (indels) were identified using a modified version of Pindel^5^. To improve specificity, a number of post-processing filters were applied that required the following:

1) For regions with sequencing depth <200X, mutant variant must be present in at least 8% of total reads.

2) For regions with sequencing depth >=200X, mutant variant must be present in at  least 4% of total reads.

3) The region with the variant should have <= 9 small (<4 nucleotides) repeats.

4) The variant is not seen in any reads in the unmatched normal sample or the unmatched normal panel.

5) The number of Pindel calls in the tumor sample is greater than 4 and either:

a. The number of mutant reads mapped by BWA in the tumor sample is greater than 0 or

b. The number of mutant reads mapped by BWA in the tumor sample is equal to 0 but there are no repeats in the variant region and there are reads mapped by Pindel in the tumor sample on both the positive and negative strand.

6) Pindel ‘SUM-MS’ score (sum of the mapping scores of the reads used as anchors) >=150

Additional unmatched normal filtering was performed using a set of unmatched normal samples (n=16). Mutations that were detected in anyone of the unmatched normal normal panel were excluded.

Variant annotation was done based on Ensembl v74 using VAGrENT.

For both substitutions and indels, variants that may have failed post processing filtering criteria but mapped to recurrent oncogenic mutations in COSMIC^6^ were retained for manual curation.

Cross referencing with known myeloma datasets

Calls retained after applying the above filters were additionally annotated with variants from MMRF CoMMpass^7^ Interim Analysis 9 exomes (n=889) and published targeted sequencing data (n=418).^8^ Calls were annotated if present at the exact genomic position with the exact mutation of if present in close proximity of a mutation (+-9 bp). All calls retained were manually curated independently by 4 individuals and the consensus was included in the results.

Structural rearrangements

Given the smaller fragment insert sizes in targeted capture, the 126 bp paired-end reads were trimmed to 50bp from the 3’ end of the read for better discovery of in structural rearrangements. Alignment on the trimmed reads was performed as previously described and structural rearrangements were detected by an in house algorithm, BRASS [https://github.com/cancerit/BRASS], which first groups discordant read pairs that span the same breakpoint and then using Velvet de novo assembler^9^ performs local assembly within the vicinity to reconstruct and determine the exact position of the breakpoint to nucleotide precision. All calls having supported by less than 5 reads were excluded. Additionally, translocations in which either of the break-points is involved with the IGH locus and all deletions, inversions and tandem-duplications involving the IGH locus were excluded for downstream analysis.

Additionally, an orthogonal pipeline using Delly^10^ (Version: 0.7.6) was used to identify structural rearrangements. Delly was run on each tumor sample using an unmatched control sample and only those calls classified as “PASS” by Delly were retained. All calls identified in the unmatched normal were also filtered. Additionally, for translocations, only those calls having at least 1 spanning read and 1 junction read or at least 4 spanning reads were retained. As previously described for BRASS, translocations in which either of the break-points is involved with the IGH locus and all deletions, inversions and duplications involving the IGH locus were excluded for downstream analysis. All calls from Delly and BRASS were further filtered for false positives using average MAPQ, CIGAR Match length and number of reads supporting the SV. MAPQ filter is the average mapping quality of all the reads supporting the SV. The value that was used as the threshold was atleast 30. CIGAR Match length is the average match length in the CIGAR string of all the reads supporting the SV. We used a threshold of 60 for CIGAR MATCH length. Lastly, supporting reads is the number of reads supporting the SV. The threshold used for support is at least 30 reads.

The resulting calls retained after the described filters were manually curated.

Copy Number Aberrations

CNVKit^11^ was used to identify somatic copy number aberrations in the data. To negate sample specific biases in CNV analysis, all 16 control samples were combined into a pooled reference. Each tumor sample is then compared with the pooled reference to identify somatic Copy Number Aberrations (CNA) in each sample. CNVKit corrects for biases in regional coverage and GC content, according to the given reference before calculating the log-ratios between the built pooled reference and tumor. Subsequently, Circular Binary Segmentation (CBS) algorithm is applied to obtain the log2fold change values.

Plots of genome-wide log2fold change values for each patient were used as basis for manual identification of CNVs.

**References**

1 Li, H. Aligning sequence reads, clone sequences and assembly contigs with BWA-MEM. *arXiv preprint arXiv:1303.3997* (2013).

2 Jones, D. *et al.* cgpCaVEManWrapper: simple execution of CaVEMan in order to detect somatic single nucleotide variants in NGS data. *Current protocols in bioinformatics* **56**, 15.10. 11-15.10. 18 (2016).

3 Nik-Zainal, S. *et al.* The life history of 21 breast cancers. *Cell* **149**, 994-1007 (2012).

4 Menzies, A. *et al.* VAGrENT: Variation annotation generator. *Current protocols in bioinformatics* **52**, 15.18. 11-15.18. 11 (2015).

5 Raine, K. M. *et al.* cgpPindel: identifying somatically acquired insertion and deletion events from paired end sequencing. *Current protocols in bioinformatics* **52**, 15.17. 11-15.17. 12 (2015).

6 Forbes, S. *et al.* The catalogue of somatic mutations in cancer (COSMIC). *Current protocols in human genetics* **57**, 10.11. 11-10.11. 26 (2008).

7 Lonial, S. *et al.* (Am Soc Hematology, 2014).

8 Bolli, N. *et al.* Analysis of the genomic landscape of multiple myeloma highlights novel prognostic markers and disease subgroups. *Leukemia*, doi:10.1038/s41375-018-0037-9 (2018).

9 Zerbino, D. R. & Birney, E. Velvet: algorithms for de novo short read assembly using de Bruijn graphs. *Genome Res* **18**, 821-829, doi:10.1101/gr.074492.107 (2008).

10 Rausch, T. *et al.* DELLY: structural variant discovery by integrated paired-end and split-read analysis. *Bioinformatics* **28**, i333-i339 (2012).

11 Talevich, E., Shain, A. H., Botton, T. & Bastian, B. C. CNVkit: Genome-Wide Copy Number Detection and Visualization from Targeted DNA Sequencing. *PLoS Comput Biol* **12**, e1004873, doi:10.1371/journal.pcbi.1004873 (2016).
